# NAFLD: From Mechanisms to Therapeutic Approaches

**DOI:** 10.3390/biomedicines10071747

**Published:** 2022-07-20

**Authors:** Karim Gariani, François R. Jornayvaz

**Affiliations:** 1 Service of Endocrinology, Diabetes, Hypertension and Nutrition, Geneva University Hospitals, Rue Gabrielle-Perret-Gentil 4, 1205 Geneva, Switzerland; 2 Diabetes Center, Geneva University, Rue Michel-Servet 1, 1206 Geneva, Switzerland; 3 Department of Cell Physiology and Metabolism, Faculty of Medicine, University of Geneva, 1211 Geneva, Switzerland

Nonalcoholic fatty liver disease (NAFLD) now represents the most frequent chronic liver disease worldwide. The severity of NAFLD ranges from simple steatosis to cirrhosis and ultimately hepatocellular carcinoma. Nonalcoholic steatohepatitis (NASH) is an intermediary step characterized by inflammation and hepatocyte injury, with or without fibrosis. NAFLD and NASH are often associated with insulin resistance, which represents a key step in the development of type 2 diabetes [1]. Other factors such as genetic predisposition, lifestyle factors, hepatic iron, leptin, antioxidant deficiencies, and intestinal dysbiosis are potential contributing factors. NAFLD is characterized by hepatocellular triglyceride accumulation in the absence of excessive alcohol consumption [2]. The topic is important and clinically relevant; type 2 diabetes is projected to increase drastically, affecting 578 million people worldwide by 2030. NAFLD and NASH are estimated to affect up to 70% of people with type 2 diabetes. The estimation of NASH prevalence is more difficult to accurately determine because the diagnosis requires a liver biopsy, which is infrequently performed. However, hepatic fibrosis is the only histologic feature of NASH independently associated with long-term overall mortality, liver transplantation and liver-related mortality. Therefore, it is of crucial importance to better understand the mechanisms leading to the development of NAFLD/NASH and fibrosis in order to find therapeutic targets to prevent fibrosis initiation or reverse this process. Moreover, NAFLD is projected to become the most common indication leading to liver transplantation in the United States in coming years.

Interestingly, in the development of NAFLD, some lipid intermediates are more likely to cause hepatic insulin resistance than others. While triglycerides are usually considered inert, other lipids such as diacylglycerols and ceramides have been clearly involved in the development of insulin resistance. Both diacylglycerols and ceramides interact with insulin signaling. Ceramides inhibit Akt2 phosphorylation and downstream insulin signaling. Diacylglycerols activate protein kinase Cε, a key pathway responsible for causing NAFLD-associated hepatic insulin resistance.

The identification of patients who might be at an increased risk of adverse outcomes is critical, since it is not feasible to screen all patients with suspected NAFLD. The main risk factors for NAFLD are obesity and diabetes, as well as other features of metabolic syndrome, including high insulin resistance, dyslipidemia, and high blood pressure. Therefore, patients with these characteristics should be screened for NAFLD. The main problem at present is making primary care physicians aware of this ever-increasing hepatic disease.

However, despite some interesting developments, no pharmacological treatments have been licensed to date, and lifestyle interventions are still crucial.

This Special Issue, entitled “NAFLD: From Mechanism to Therapeutic Approaches” is focused on the pathophysiology of NAFLD, new biomarkers to detect NAFLD, future potential therapeutic opportunities to treat and counteract the evolution of this disorder, and novel preclinical models. This Special Issue includes a total of 19 research papers, 19 review articles, and 2 systematic reviews.

In this Editorial, we would like to highlight, firstly, a paper that strengthens the positive impact of exercise on NAFLD. As mentioned, there is currently no available specific treatment to target NAFLD/NASH. Thus, lifestyle measures should be encouraged, and consequently physical activity should be carefully considered. Indeed, sedentary behavior is now a pandemic health problem contributing to the pathophysiology of obesity and type 2 diabetes. Moreover, sedentarity is further associated with liver disease and particularly with NAFLD/NASH. Insulin resistance is potentially a link between sedentarity and NAFLD/NASH. Insulin resistance is closely related to ectopic lipid deposition, i.e., “fat in the wrong place”, most notably in the liver, but also in the skeletal muscle. As skeletal muscle accounts for 60 to 80% of insulin stimulation-mediated glucose metabolism, rising skeletal muscle activity by means of physical exercise constitutes a promising therapeutic approach. To support this, there is evidence that physical activity can impact different molecular pathways in a positive manner, for example, via AMP-activated protein kinase and insulin signaling, glucose transporter 4 (GLUT4) translocation, and the modulation of insulin action, cellular substrate flow, ectopic lipid, and glycogen storage [3]. From a pathophysiological point of view, therefore, there is evidence that physical exercise could lead to substantial clinical benefits in persons with type 2 diabetes and/or NAFLD/NASH. However, we currently lack long-term observational studies, as a key problem may be the motivation of patients in the long term. Therefore, it is of crucial importance to integrate physical activity into everyday life, for example, by taking the stairs instead of the elevator, or leaving the bus one stop prior to the usual stop. These small modifications maintained in the long term could help in reversing insulin resistance, notably in skeletal muscle, and avoid novo lipogenesis, therefore improving NAFLD/NASH.

NAFLD remains difficult to diagnose, especially in the early phase of the disease. New non-invasive tools are therefore required for the diagnosis of steatosis and fibrosis, allowing a reliable identification of individuals at risk who can be referred to hepatologists. Several papers published in this issue discuss the potential role of various approaches to detect NAFLD non-invasively and assess its severity, including machine-learning-based or combined screening tools such as plasmatic microRNAs and imaging [4,5,6,7,8]. The implementation of comprehensive screening programs is urgently needed to improve the management of this disorder and unexpensive non-invasive screening tests may have a significant positive impact on this aspect.

An important number of preclinical models have already contributed to improving our understanding of the complex pathophysiology of NAFLD; however, these models do not always exactly mimic human NAFLD, and therefore are not always ideal for addressing a specific research question. Consequently, new reliable models displaying identical liver phenotypes to humans are needed. In this issue, interesting novel preclinical models are evaluated, including a guinea pig disease model, chimeric mice with humanized livers, and a novel 2-hit zebrafish model to specifically model the early pathogenesis of NAFLD [9,10,11].

Pursuing research efforts in the field of NAFLD will improve our understanding of its complex pathogenesis and may potentially elicit the development of new screening tools and new therapeutic approaches.

We would like to warmly thank all scientists who participated in peer reviews, as well as the managing editor of this Special Issue of *Biomedicines*.
